# Testing the Ingroup Advantage in Emotion Perception from Dynamic Posed and Spontaneous Expressions

**DOI:** 10.1007/s10919-025-00492-1

**Published:** 2025-08-22

**Authors:** Yong-Qi Cong, Lidya Yurdum, Agneta Fischer, Disa Sauter

**Affiliations:** University of Amsterdam, Amsterdam, Netherlands

**Keywords:** Facial expressions, Emotion recognition, Perception, Spontaneous, Ingroup advantage, Cross-cultural

## Abstract

The ingroup advantage refers to the phenomenon of emotions being more accurately recognised when expressors and perceivers share the same cultural background. Though well-documented in studies using posed facial expressions, whether the in-group advantage generalizes to spontaneous expressions remains unclear. This pre-registered study examined cross-cultural emotion recognition using dynamic posed and spontaneous expressions in a balanced design. Perceivers from the Netherlands (*N* = 762) and China (*N* = 738) judged emotions from facial expressions produced by Dutch and Chinese expressors in a forced-choice emotion recognition task. Contrary to our hypothesis, we did not find a mutual ingroup advantage. Instead, we found a Decoder and an Encoder effect for both posed and spontaneous expressions. Specifically, Dutch Perceivers outperformed Chinese Perceivers in recognising both Dutch and Chinese emotional expressions (the Decoder effect), while Chinese emotional expressions were better recognised than Dutch expressions by both Chinese and Dutch Perceivers (the Encoder effect). Bayesian analyses confirmed robust evidence for these effects. These findings challenge the robustness of the ingroup advantage and highlight the importance of using ecologically valid stimuli in the study of nonverbal emotional communication.

**Supplementary Information:**

The online version contains supplementary material available at 10.1007/s10919-025-00492-1.

## Introduction

Culture has consistently been shown to influence how emotions are perceived from non-verbal expressions (Fang et al., 2021; Kawahara et al., 2021; Matsumoto, 1992; Wood et al., 2016). Specifically, people are often better at recognising emotions from facial expressions when the perceivers’ cultural background matches that of the expressors. This phenomenon has been termed the *ingroup advantage* (Elfenbein & Ambady, 2002). However, existing research has predominantly used posed emotional expressions taken from standardised stimuli sets. The current study aims to test the generalisability of the ingroup advantage effect by using spontaneous facial expressions as stimuli and comparing them directly to posed expressions.

The vast majority of research on emotion perception has relied on posed facial expressions (Dawel et al., 2022). Such expressions, while useful for controlled experiments, may not be representative of real-life emotional expressions that occur spontaneously (Krumhuber et al., 2021; Namba et al., 2017). For example, posed and spontaneous smiles differ in their amplitude, timing and appearance (Cohn & Sshmidt, 2004; Park et al., 2020; Schmidt et al., 2009). Such differences in posed and spontaneous expressions could influence how they are perceived. Indeed, empirical evidence shows that perceptions of spontaneous expressions can differ substantively from how we see posed expressions (Cong et al., 2025; Kohler et al., 2010; Naab & Russell, 2007), leading to divergent conclusions depending on whether posed or spontaneous expressions are used as stimuli. For example, Davis and Gibson (2000) replicated previous findings of emotion recognition deficits in schizophrenia only when using posed expressions, while the schizophrenia group performed better than both control groups when spontaneous expressions were used. Another example comes from a recent study investigating whether negative emotions were better recognised than positive emotions, Cong and colleagues (2025) found this to be the case only when using posed expressions, while the pattern of results reversed for judgments of spontaneous emotional expressions. These findings raise questions about the generalisability of existing theories based on findings from studies using posed expressions.

Here, we are particularly interested in the question of whether findings from cross-cultural emotion recognition studies based on posed expressions extend to spontaneous expressions, given theoretical and empirical differences between the two types of stimuli. Understanding the role of culture in emotion perception in everyday contexts requires testing whether established phenomena, such as the ingroup advantage effect, hold true when spontaneous expressions are used.

The ingroup advantage refers to the observation that non-verbal emotional expressions are better recognised when the expressor and perceiver share the same cultural background, compared to when their cultural backgrounds differ (Elfenbein & Ambady, 2002; Laukka & Elfenbein, 2021). This phenomenon is theorised to arise from subtle differences in how emotions are expressed non-verbally in different cultures, known as *emotion dialects* (Elfenbein, 2013; Elfenbein et al., 2007). When perceivers are less familiar with the emotion dialect of the expressor, emotion recognition is impaired (Elfenbein & Ambady, 2003). A substantial body of research supports the ingroup advantage in cross-cultural emotion recognition. However, the reliance on posed expressions in most of this work leaves open the question of whether the effect extends to spontaneous expressions.

Only a few studies have directly tested the ingroup advantage using spontaneous facial expressions, and they have yielded inconclusive results (Fang & Ge, 2024; Kang & Lau, 2013; Matsumoto et al., 2009). One study by Matsumoto and colleagues (2009) examined American and Japanese observers’ judgements of spontaneous expressions produced by athletes at the completion of medal matches from the Olympic Games. They did not find an ingroup advantage. However, the ingroup and outgroup comparison was not exactly matched: A total of 15 “American” expressions were used as ingroup stimuli for the American perceivers and as outgroup stimuli for the Japanese perceivers, but of those, only 3 expressions were produced by American athletes; the remaining 12 expressions were produced by White athletes of other nationalities. This means that part of the “ingroup” stimuli for the American perceivers were arguably produced by outgroup members. Moreover, all the expressions used as stimuli were preselected to match expressions based on theoretical prototypes using the Facial Action Coding System (FACS). FACS-standardising the expressions ensures that the ingroup and outgroup expressions for the same emotion share the same facial muscle movements. As a result, the potential for *emotion dialects* to emerge is limited. Nevertheless, this study was the first to use spontaneous expressions and its results challenged the existence of an ingroup advantage.

To test whether stimulus type (posed versus spontaneous) influences the ingroup advantage effect, it is particularly useful to directly compare the perception of posed with spontaneous expressions. This was done in a study by Kang and Lau (2013), which used spontaneous expressions from recordings of interviews where White American and Asian American participants recalled life experiences. Emotion perception from these videos was compared to judgments of posed expressions from the JACFEE stimulus set (Biehl et al., 1997). The authors found an ingroup advantage in emotion perception from spontaneous, but not posed, expressions. These results suggest that the presence of an ingroup advantage may depend on whether posed or spontaneous expressions are used as stimuli. However, their finding that there was no ingroup advantage for posed expressions is surprising in light of prior research (Elfenbein & Ambady, 2003). Also, although Kang and Lau’s (2013) study directly compared posed and spontaneous expressions, the two conditions differed in several ways, which make it challenging to draw strong conclusions from their findings. Firstly, the number of emotions as well as the number of total expressions in the posed and spontaneous conditions differed: Perceivers viewed 48 unique expressions representing 6 emotions in the posed expressions condition, but only 14 unique expressions representing 5 emotions in the spontaneous expressions condition, of which just 3 emotions were the same as the ones used in the task with posed expressions. The specific emotions are important because the magnitude of the ingroup advantage has been found to differ across emotions (Elfenbein & Ambady, 2002). The difference observed between the posed and spontaneous expressions may therefore (at least partly) be attributed to the different emotion categories. Moreover, the posed expressions were taken from the JACFEE, which is a set of facial expressions that are FACS-standardised, such that the facial muscle movements for each emotion map on to theoretical prototypes. The expressions for each emotion are therefore identical on the Asian and White faces. This removes any potential emotion dialects in the posed expressions, which could be why the ingroup advantage was not found using these stimuli.

Contrary to the conclusions of Matsumoto and colleagues (2009) and Kang and Lau (2013), a recent study investigating cross-cultural emotion perception of posed and spontaneous expressions did find an ingroup advantage for judgments of both posed and spontaneous expressions. Fang and Ge (2024) examined Canadian and Chinese perceivers’ recognition of expressions of anger and disgust by Canadian and Chinese expressors. The posed expressions were created by instructing expressors to show anger and disgust so that friends would understand how they felt. The spontaneous expressions were created by expressors recalling events from their life that had made them feel angry or disgusted. One important methodological improvement of this study was that the researchers used dynamic facial expressions as stimuli. Dynamic facial expressions are more representative of real-life emotional expressions and the dynamic aspects of facial expressions have been shown to influence how facial expressions are perceived (Krumhuber et al., 2013). With dynamic stimuli, the ingroup advantage was observed for both posed and spontaneous facial expressions. Though it only examined two emotions, this study provides initial evidence that there may be an ingroup advantage in emotion perception from both posed and spontaneous facial expressions, if ecologically valid stimuli are used where emotion dialects have the possibility to occur.

### The Current Study

Existing evidence on the presence of an ingroup advantage using spontaneous expressions is thus inconclusive. To test whether the ingroup advantage hypothesis can be generalised to spontaneous expressions, the current study investigated cross-cultural emotion recognition from spontaneous expressions in comparison to posed expressions. We carefully controlled for several potential confounding variables. Specifically, we examined cross-cultural emotion perception using a large set of dynamic facial expressions produced by Dutch and Chinese individuals. To ensure maximal comparability between the two conditions, we used posed and spontaneous expressions produced by the same expressors. A balanced set of four positive and four negative emotions were included in order to allow for generalisable conclusions across emotions. In a fully balanced design, we compared emotion perception from posed and spontaneous facial expressions, with Dutch and Chinese perceivers judging emotional expressions from their own, as well as the other, culture. Our central hypothesis was that there would be an ingroup advantage in emotion perception from both posed and spontaneous facial expressions. The study was pre-registered at https://osf.io/3vb2p/?view_only=be22fc3b2732452ea6eaa5abf3a71830 prior to data collection^1^.

## Method

### Materials

The stimuli were taken from a video corpus of dynamic posed and spontaneous facial expressions produced by Chinese and Dutch individuals (Cong et al., 2025). The expressors were born and raised in their respective countries and had not lived abroad for more than three months. Expressions of eight emotions were included: anger, disgust, fear, sadness, joy, compassion, love, and pride. For the translations of each emotion label see Supplementary Table 1.

All stimuli were short, colour video clips capturing the head and upper torso of an expressor. To elicit spontaneous expressions, individuals were asked to recall events from their own life during which they had experienced each of the target emotions. They were asked to retell their emotional experience as clearly and in as much detail as possible, so that another person could understand exactly how they had felt. Expressors then rated the extent to which they had experienced the target emotion during the recall. After the recording session was completed, expressors were asked to review their video recordings and, for each emotion, select a section of the video (maximally five seconds in length) that they thought best represented their expression of that emotion. This could be either a fragment during which they were speaking or a moment of silence. The videos were then trimmed to the selected portion by the researchers to create the spontaneous expression stimuli. Posed expressions were gathered after the spontaneous expressions were recorded but before the expressors selected their most representative spontaneous expressions. For the posed expressions, expressors were asked to show, without speaking, how they would express each target emotion to someone they know. They were allowed to make multiple attempts until they were satisfied with the expression they posed. The order in which emotions were elicited was randomised within each subject for the production of spontaneous expressions, and again randomised for the production of posed expressions. All videos were muted.

Expressions that were not recognised at above-chance level by within-cultural perceivers (Cong et al., 2025) were not included in this study. This resulted in a total of 267 Dutch posed expressions, 207 Dutch spontaneous expressions, 255 Chinese posed expressions, and 147 Chinese spontaneous expressions. The full list of stimuli with the corresponding emotions can be found here: https://osf.io/mfu5j/.

### Participants

Participants for the perceptual judgment study were recruited using the online recruitment service Cint. Individuals who were born and raised in their respective country and who had not lived abroad for more than six months were eligible to participate. Sample size was determined a priori based on a power analysis using the average effect size of the ingroup advantage (9%) obtained from a meta-analysis (Elfenbein & Ambady, 2002). To achieve statistical power of 0.8 for a two-sample *t*-test (R package *pwr*), a total of 1526 participants were needed, corresponding to 763 participants per culture. A total of 877 adults from the Netherlands (431 women, 441 men, and 5 identified as other) and 825 adults from China (417 women and 408 men) participated in the study. Participants received monetary compensation for their participation.

We pre-registered the exclusion of participants whose overall performance was two or more standard deviations lower than the average accuracy of the participants in their country, or whose average response time was two or more standard deviations below the mean of participants in their country. No one was excluded based on response time; 23 Dutch and 21 Chinese participants were excluded due to poor performance. To ensure good data quality in this online study, we applied an additional exclusion criterion to detect participants who likely did not watch the videos. Because most of the videos were more than one second long, the participant likely did not watch the video before answering, if a trial was finished in less than one second. Those who completed a high proportion (> 50%) of trials in less than one second were excluded. This led to the exclusion of an additional 91 Dutch and 64 Chinese participants. After exclusions, the remaining sample consisted of 1500 adults between the ages of 18 and 85 years ($\:{M}_{age}$ = 30.57, $\:S{D}_{age}$ = 8.36)^2^, including 738 Chinese ($\:{M}_{age}$ = 34.10, $\:S{D}_{age}$ = 8.49) and 762 Dutch ($\:{M}_{age}$ = 27.14, $\:S{D}_{age}$ = 6.64) participants.

### Design and Procedure

The stimuli were divided into four subsets, grouped by expressor culture (Chinese or Dutch) and expression type (posed or spontaneous). For each participant, a semi-random set of 16 expressions from each of the four subsets was drawn. The draw was semi-random such that each of the eight emotions was represented twice within the 16 stimuli per subset. Each participant thus saw a total of 64 videos, including posed and spontaneous expressions of all eight emotions and from both cultures (i.e., all conditions were manipulated within-subject).

The online study was administered using Gorilla Experiment Builder (Anwyl-Irvine et al., 2020). Instructions were in Dutch for participants in the Netherlands and in Mandarin Chinese for participants in China. Participants were instrucet ted to sit in a quiet room with no distractions. They were encouraged to finish the study in one sitting, with a short borilla Experiment Bureak half-way; they were required to finish the study within 24 h before their session expired.

After giving informed consent, participants first answered demographic questions about their age, gender, and birthplace, and whether they had lived abroad. Those who met the screening criteria were then directed to the emotion recognition task. Participants were able to watch each video clip as many times as they wanted before answering a forced-choice question to indicate which emotion they thought was being expressed. The response options corresponded to the eight emotion categories anger, disgust, fear, sadness, joy, compassion, love, and pride. The task auto-progressed to the next trial when a response had been selected.

## Results

Our hypothesis was that there would be an ingroup advantage effect for both posed and spontaneous expressions, such that emotion recognition would be better when the culture of the perceiver matched that of the expressor (Elfenbein & Ambady, 2002; Laukka & Elfenbein, 2021). This can be operationalised in two ways (Kang & Lau, 2013). The first approach is to keep the stimulus culture (i.e., expressors) constant and compare the recognition accuracy of the two perceiver groups (e.g., comparing Dutch perceivers’ performance to Chinese perceivers’ performance for Dutch expressions). The second method is to keep the perceiver group constant and compare the recognition accuracy of the two stimulus sets within one perceiver group (e.g., comparing the recognition rates for Chinese expressions with those of Dutch expressions, both judged by Chinese perceivers). We tested our hypothesis using both approaches.

We first calculated each perceiver’s accuracy per Expression Type and Expressor Culture (see Supplementary Materials for the confusion matrices). We then fit a linear mixed-effects model to the data, regressing accuracy onto Expression Type (posed vs. spontaneous), Expressor Culture (Chinese vs. Dutch), and Perceiver Culture (Chinese vs. Dutch). Expressor ID was included as a random effect, which allowed us to account for the repeated-measures nature of the data by including random intercepts for participants^3^. We then ran a Type I ANOVA on the fitted model to obtain F-tests with Satterthwaite-approximated degrees of freedom. This revealed significant main effects of Perceiver Culture (*F*(1, 1498) = 32.03, *p* < .001), Expressor Culture (*F*(1, 4494) = 98.97, *p* < .001), and Expression Type (*F*(1, 4494) = 1086.09, *p* < .001), alongside two-way interactions between Perceiver and Expressor Culture (*F*(1, 4494) = 18.71, *p* < .001), between Expressor Culture and Expression Type (*F*(1, 4494) = 14.82, *p* < .001), and between Perceiver Culture and Expression Type (*F*(1, 4494) = 20.39, *p* < .001).

The interactions with Expression Type indicated that emotion recognition differs between posed and spontaneous expressions. Therefore, we tested our hypotheses regarding the ingroup advantage for posed and spontaneous expressions separately.

### Posed Expressions

Regressing accuracy (for posed expressions only) onto Expressor and Perceiver Culture, with Perceiver ID as a random effect, revealed main effects of both Expressor Culture (*F*(1, 1498) = 83.96, *p* < .001) and Perceiver Culture (*F*(1, 1498) = 40.23, *p* < .001), as well as an interaction between them (*F*(1, 1498) = 16.93, *p* < .001; F-tests conducted with Satterthwaite-approximated degrees of freedom). We then ran follow-up linear regressions testing for an ingroup advantage using the two approaches described above.

*Holding Expressor Culture Constant*.

First, we held the culture of the Expressor constant and compared the performance of the Perceiver groups. An ingroup advantage would entail Dutch Perceivers outperforming Chinese Perceivers with Dutch expressions, and Chinese Perceivers outperforming Dutch Perceivers with Chinese expressions. Splitting the data by Expressor Culture, we ran linear models in which we regressed accuracy on Perceiver Culture (with Chinese stimuli set as the intercept).

**Fig. 1:**
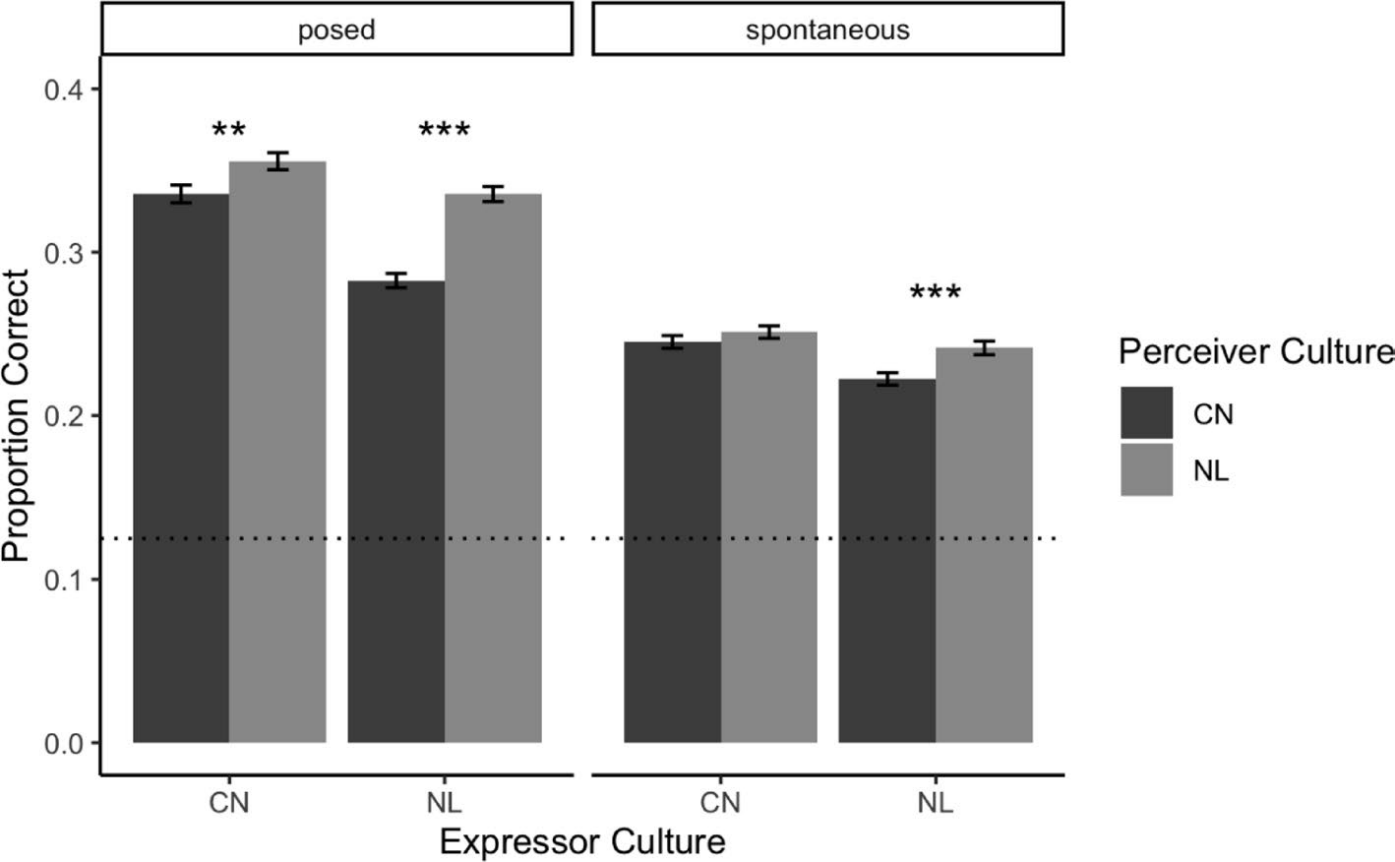
Compared to Chinese Perceivers (dark grey bars), Dutch Perceivers (light grey bars) more accurately inferred the emotions expressed in posed Dutch and Chinese expressions (left panel). Dutch perceivers performed better than Chinese perceivers for spontaneous Dutch expressions (right panel), but no difference was observed between the groups for spontaneous Chinese expressions. The bars indicate recognition accuracy as a proportion of correct responses across all trials, averaged across participants. Error bars reflect standard errors. The dotted line denotes chance-level recognition (1/8 = 0.125). Significance stars refer to the mixed linear regressions reported in the text. *** *p* < .001, ** *p* < .01, * *p* < .05

Contrary to our hypothesis, we did not find a mutual ingroup advantage for posed emotional expressions. Instead, we found that Dutch Perceivers outperformed Chinese Perceivers when viewing both Dutch expressions ($\:\:{\beta\:}_{Dutch\:Perceivers}$ = 0.053, 95% CI [0.04, 0.07], *p* < .001), and Chinese expressions ($\:{\beta\:}_{Dutch\:Perceivers}$ = 0.02, 95% CI [0.01, 0.03], *p* = .008; Fig. 1). The interaction effect reflected the fact that while Dutch Perceivers’ recognition accuracy was higher than that of Chinese Perceivers for both Dutch and Chinese expressions, the difference between the two Perceiver cultures was larger for Dutch expressions (for group means see Supplementary Materials). This means that following the first approach (i.e., holding Expressor Culture constant while comparing performance between Dutch and Chinese Perceivers), we found an ingroup advantage with Dutch expressions, but not with Chinese expressions.

The finding that Dutch Perceivers outperformed Chinese Perceivers when viewing Chinese expressions contrasts with our hypothesis of an ingroup advantage. We therefore evaluated the relative support in the observed data for the original hypothesis (Chinese Perceivers $\:>$ Dutch Perceivers) versus the null (Chinese Perceivers $\:\le\:$ Dutch Perceivers) using Bayesian inference for informative hypotheses. We used the R package *bain*, version 0.2.11 (Hoijtink et al., 2019), which uses the Bayes factor to evaluate the support for hypotheses in a wide range of models. A Bayes factor quantifies this support in terms of the relative likelihood of one hypothesis over the other. For example, $\:B{F}_{1}=3\:$means the data are 3 times more likely under the alternative hypothesis than the null hypothesis. We found extremely low support for the alternative hypothesis (Chinese Perceivers $\:>$ Dutch Perceivers) compared to the null ($\:B{F}_{1}$ = 0.004). The Bayes factor in favour of the null can also be expressed as the reciprocal of $\:B{F}_{1}$. In this case, we have very strong evidence in favour of the null hypothesis ($\:B{F}_{0}=\:1\:/\:B{F}_{1}=$ 245.08) over the ingroup advantage hypothesis.

#### Holding Perceiver Culture Constant

Next, we tested whether there is an ingroup advantage following the second approach frequently used in the literature. The second approach holds Perceiver Culture constant while comparing the recognition accuracy of expressions from different Expressor groups. In this approach, an ingroup advantage would entail Chinese Perceivers performing better with Chinese expressions than Dutch expressions, and Dutch Perceivers performing better with Dutch expressions than Chinese expressions.

**Fig. 2:**
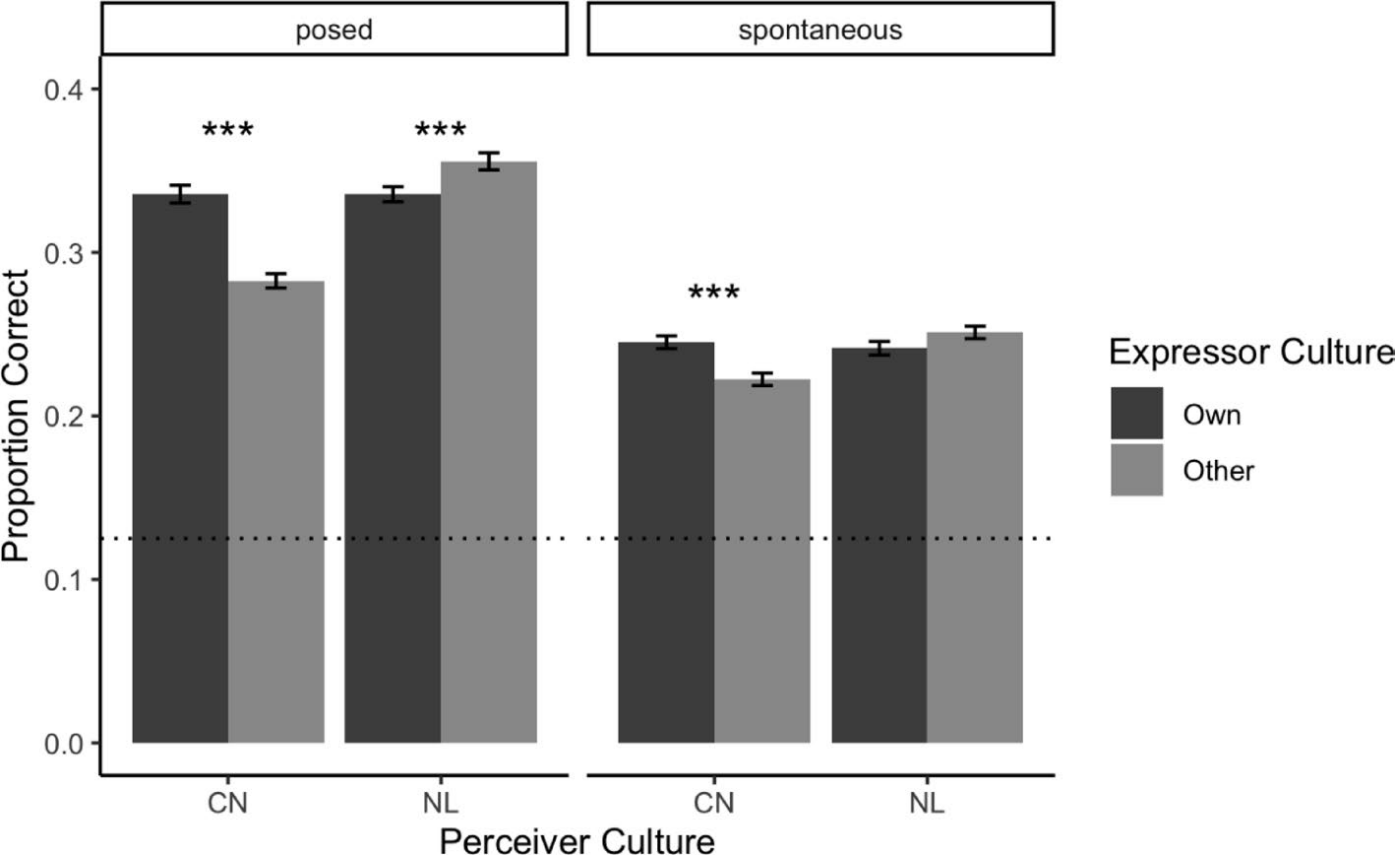
Chinese Perceivers demonstrated an advantage on stimuli from their own culture (dark grey bars), regardless of stimuli type. Dutch Perceivers, by contrast, did not demonstrate an own-culture advantage, and displayed the opposite pattern in posed stimuli: they more accurately inferred the emotions in Chinese expressions than Dutch expressions. The bars indicate recognition accuracy as a proportion of correct responses across all trials, averaged across participants. Error bars reflect standard errors. The dotted line denotes chance-level recognition (1/8 = 0.125). Significance stars refer to the mixed linear regressions reported in the text. *** *p* < .001, ** *p* < .01, * *p* < .05

Again, we did not find a mutual ingroup advantage using this method. Instead, we found that emotions were more accurately recognised from Chinese expressions than from Dutch expressions by both Chinese ($\:{\beta\:}_{Dutch\:Expressions}$ = -0.05, 95% CI [-0.07, -0.04], *p* < .001) and Dutch Perceivers ($\:{\beta\:}_{Dutch\:Expressions}$ = -0.02, 95% CI [-0.03, -0.01], *p* = .004). This means that, in contrast to the ingroup advantage hypothesis, Perceivers were not consistently more accurate when viewing expressions from their own culture (see Fig. 2). Rather, the Chinese posed expressions were overall better recognised, regardless of Perceiver Culture.

### Spontaneous Expressions

To investigate the ingroup advantage in the recognition of spontaneous expressions, we followed the same analytical approach as for posed expressions. Regressing accuracy (for spontaneous expressions only) onto Expressor and Perceiver Culture, with Perceiver ID as a random effect, revealed main effects of both Expressor Culture (*F*(1, 1498) = 21.62, *p* < .001) and Perceiver Culture (*F*(1, 1498) = 8.42, *p* = .004), but no interaction between the two (*F*(1, 1498) = 3.5, *p* = .06; F-tests conducted with Satterthwaite-approximated degrees of freedom). We thus did not observe a mutual ingroup advantage for the spontaneous expressions. We then ran follow-up linear regressions testing for an ingroup advantage using the same two approaches described above.

#### Holding Expressor Culture Constant

First, we compared the performance of the Perceiver groups when viewing stimuli from the same Expressor Culture. Dutch Perceivers outperformed Chinese Perceivers when viewing Dutch expressions ($\:{\beta\:}_{Dutch\:Perceivers}$ = 0.02, 95% CI [0.01, 0.03], *p* < .001), with the data providing strong evidence in favour of an ingroup advantage for Dutch expressions ($\:B{F}_{1}=2841.07)$. However, when viewing Chinese expressions, recognition accuracy levels of Dutch and Chinese Perceivers did not significantly differ from each other ($\:{\beta\:}_{Dutch\:Perceivers}$ = 0.01, 95% CI [-0.004, 0.02], *p* = .27). The data provided moderate support for the null hypothesis over the ingroup advantage hypothesis: Chinese Perceivers do not perform better than Dutch Perceivers when viewing Chinese expressions ($\:B{F}_{0}=6.31)$. This means that when holding Expressor Culture constant, we found an ingroup advantage for Dutch expressions, but not for Chinese expressions.

#### Holding Perceiver Culture Constant

Next, we split the data by Perceiver Culture and compared performance across expressor culture. We found that Chinese Perceivers were better at recognising emotions from Chinese expressions than from Dutch expressions ($\:{\beta\:}_{Dutch\:Expressions}$ = -0.02, 95% CI [-0.03, -0.01], *p* < .001), whereas the Dutch perceivers did not perform differently with regard to Chinese and Dutch expressions ($\:{\beta\:}_{Dutch\:Expressions}$ = -0.01, 95% CI [-0.02, 0.001], *p* = .08). Bayesian analyses yielded evidence for an ingroup advantage for Chinese Perceivers ($\:B{F}_{1}=59521.68)$, but evidence against an ingroup advantage for Dutch Perceivers ($\:B{F}_{0}=22.92)$.

To summarise, we did not find a mutual ingroup advantage for either posed or spontaneous expressions. In both approaches of testing (holding Perceiver culture constant or holding Expressor culture constant), there was only evidence for an ingroup advantage in half of the data. Instead, these findings point to a Decoder and an Encoder effect (Beaupré & Hess, 2005), especially for posed expressions.

## Discussion

The current study investigated the ingroup advantage in emotion perception comparing posed and spontaneous expressions. We used large samples of stimuli and perceivers and a fully balanced design, with expressions of eight emotions from the same expressors. We predicted an ingroup advantage for both posed and spontaneous expressions, meaning that perceivers would be better at recognising emotional expressions from their own culture as compared to expressions from the other culture, irrespective of whether the facial expressions were posed or spontaneous. Contrary to our hypothesis, we did not find a mutual ingroup advantage. Instead, we found an Encoder and a Decoder effect (Beaupré & Hess, 2005): Dutch perceivers were better at recognising Dutch, but also Chinese, expressions compared to the Chinese perceivers. On the other hand, Chinese expressions were better recognised than Dutch expressions by perceivers from both cultures. Below, we discuss the methodological and theoretical implications of these findings.

### Understanding the Encoder and Decoder Effect

We found evidence of a Decoder effect, with Dutch perceivers outperforming Chinese perceivers with expressions from both cultures. This finding is consistent with some previous research on cross-cultural emotion recognition. For example, perceivers from individualistic cultures have been found to have higher emotion recognition accuracy than those from collectivistic cultures (Matsumoto, 1992; Zhang et al., 2015). Since the Dutch culture is more individualistic than the Chinese culture (Hofstede, 2011), this aligns with our finding of Dutch perceivers’ overall better performance in emotion recognition compared to Chinese perceivers.

One proposed explanation for a Decoder effect emerging when Western (i.e., Western Europeans and North Americans) perceivers are compared with (East) Asian perceivers refers to thinking styles: Westerners are more inclined to associate outward appearance with inner emotional states, while Easterners tend to perceive a gap between people’s internal states and their outward expressions (e.g., Ji et al., 2023). Relatedly, Easterners often exhibit non-dualistic or dialectical thinking (Peng & Nisbett, 1999; Yama, 2017). This refers to the thinking style that recognises opposing perspectives or situations as co-existing or complementing, instead of being mutually exclusive. The preference for non-dualistic thinking may reduce the tendency for direct one-to-one mapping between facial expressions and emotion concepts or categories. Consistent with this notion, Easterners are also more likely to perceive multiple concurrent emotions in one facial expression (Fang et al., 2019). Westerners may thus be more likely to perform better in emotion recognition tasks with facial emotion cues, given that their cultural perspective aligns more closely with the idea of convergence between facial expressions and single emotion categories. This advantage may be particularly pronounced in forced-choice emotion recognition tasks, when perceivers are forced to choose only one emotion category to match a particular facial expression. These cultural differences in the interpretation of facial expressions and general thinking styles could explain the superior performance of Dutch perceivers in our study.

In addition to the Decoder effect, we also found that the Chinese expressions were better recognised than the Dutch expressions by both groups of perceivers, which points to an Encoder effect. Previous research has highlighted several factors that can influence how well different emotional facial expressions can be recognised. One such factor is historic heterogeneity of long-history migration (Rychlowska et al., 2015), which refers to the number of source cultures that have contributed to a country’s present-day population. The higher the number of source cultures, the more historically heterogeneous a country is seen to be. Research has found that facial expressions from countries whose populations are historically more heterogeneous, compared to expressions from cultures with historically more homogenous populations, are more easily recognised (Wood et al., 2016). However, the Encoder effect observed in the current study is in the opposite direction from what would be predicted by this theory, suggesting that other variables are at play.

Apart from historic heterogeneity, differences between how well emotions from different cultures are recognised can also result from other differences between the stimuli sets. Stimuli sets can differ on other dimensions than the cultural background of the expressors. For example, the intensity of the expressions could differ such that the magnitude of muscle movements in the expressions from one culture are greater than another. Such differences in intensity are not always controlled, even in experimental studies. Nevertheless, they may have an effect on whether an ingroup advantage is observed in cross-cultural emotion recognition. This might have contributed to the Encoder effect in the current study.

#### Methodological Considerations for Studying Cross-cultural Emotion Recognition

The present study is not the first to find no evidence of an ingroup advantage for posed facial expressions. Kang and Lau (2013) did not find an ingroup advantage in emotion perception using posed expressions. Reyes et al. (2018) also did not find a mutual ingroup advantage. Their findings showed that both South Asian and White Canadians were more accurate in emotion recognition from South Asian expressions, while White perceivers performed better overall than the South Asian perceivers (Reyes et al., 2018). Our results thus align with those previous findings. An important addition of the present study is that it extends the finding of an Encoder and Decoder effect (rather than a mutual ingroup advantage) to spontaneous expressions.

In a commentary on the meta-analysis by Elfenbein and Ambady (2002), where the ingroup advantage was first set out, Matsumoto (2002) raised an important methodological concern. He argued that certain criteria must be met to effectively test the ingroup advantage effect. A key requirement is that studies should adopt a balanced design, such that perceivers from all cultures view stimuli from their own as well as other expressor groups. He argued that studies with an unbalanced design might observe a pattern of results that appears to be an ingroup advantage, when the difference between ingroup and outgroup emotion recognition is caused by something else. Our findings support this reasoning. If we had only included Chinese perceivers who viewed expressions from the two expressor cultures, we would have falsely concluded that we found evidence of an ingroup advantage, since the Chinese expressions were better recognised by the Chinese perceivers. Similarly, if we had only used Dutch expressions which were viewed by perceivers from both cultures, we would have falsely drawn the conclusion that there is an ingroup advantage based on the observation that Dutch perceivers performed better. Therefore, our study provides strong support for the idea that it is important to adopt a balanced design when studying cross-cultural emotion recognition.

We did not find the hypothesised ingroup advantage with posed or spontaneous expressions. Nevertheless, a direct comparison of recognition patterns for the two types of stimuli can be informative, as they point to more pronounced cross-cultural differences for posed, as compared to spontaneous, expressions. In particular, we found that both the Encoder and Decoder effects were more pronounced for posed than for spontaneous expressions. Dutch perceivers were better than Chinese perceivers in recognising both the Chinese and Dutch posed expressions. However, for spontaneous expressions, Dutch perceivers did not have an advantage over Chinese perceivers in judging the Chinese expressions, and their advantage in judgements of Dutch expressions was smaller compared to posed expressions. These findings highlight the importance of using spontaneous emotion expression in cross-cultural emotion recognition research, as using posed expressions could lead to over-estimates of cultural differences.

## Conclusion

In a carefully controlled study with a balanced design, we did not find evidence supporting a mutual ingroup advantage in cross-culture emotion recognition from posed or spontaneous facial expressions. These findings add to the growing body of research that challenge the robustness of this effect and point to the importance of understanding the boundary conditions under which the effect does emerge.

## Electronic Supplementary Material

Below is the link to the electronic supplementary material.

Supplementary Material 1

## Data Availability

All data supporting the reported results will be made publicly available upon publication of this manuscript. These data and analyses scripts are available via this link: https://osf.io/mfu5j/.

## Footnotes

1 We report all conditions and measures, but not all pre-registered hypotheses are discussed in this manuscript.

2 We aimed to recruit people aged between 18 and 40, but due to technical error a small portion of the sample fell outside of this age bracket. Fourteen participants were under the age of 18 and 142 participants above 40, while 1344 were within the target age range. We excluded three participants who were younger than 16, as they were below the age of consent. We then compared the emotion recognition levels of the participants outside the target age range to those above it. As no differences in recognition rates were found, we included all participants in the final analyses.

3 We pre-registered analyses with mixed logistic regressions using accuracy at the trial level as the outcome variable. The findings are consistent with the analyses reported here. These pre-registered analyses are reported in the Supplementary Materials, together with analyses per emotion.
