# Supplementary material for: Testing the Ingroup Advantage in Emotion Perception from Dynamic Posed and Spontaneous Expressions
Source: J Nonverbal Behav. 2025 Aug 22;49(4):489–503. doi: 10.1007/s10919-025-00492-1 (PMC12627183; doi:10.1007/s10919-025-00492-1)
Supplement: Supplementary file 1 — Supplementary Material 1 [file 10919_2025_492_MOESM1_ESM.pdf]

Supplementary Material

Supplementary Text

1. Preregistered Analyses using Trial-level Accuracy

In the main manuscript, we investigated the ingroup advantage using the proportion of correct responses per participant (Raw Accuracy) as the outcome measure. This approach is appropriate for the cross-emotion analyses reported in the main text, but is not well-suited for the per-emotion analyses we report in this supplementary file. Each Perceiver saw a total of 64 videos, consisting of two videos per Expression Type, Expressor Culture, and Emotion. This resulted in a limited range of accuracy per analysis cell: participants could be correct on both trials (accuracy = 1), one trial (accuracy = .5), or neither (accuracy = 0). The resulting outcome measure is therefore not continuous. For this reason, we preregistered a different analysis approach where we regress trial-level binary accuracy (correct vs. incorrect) onto the predictor variables, using mixed logistic regressions (see https://osf.io/3vb2p). The results of the preregistered analyses are consistent with the results reported in the manuscript. As in the manuscript, we investigate the ingroup advantage in two ways: first holding Expressor culture constant, and then holding Perceiver culture constant.

Following our pre-registered analysis plan, we first performed a mixed logistic regression where we regressed Recognition Accuracy (operationalised as binary trial-level outcome; correct vs. incorrect) onto Expression Type (posed vs spontaneous), Expressor Culture, Perceiver Culture, and Emotion. Perceiver ID was included as a random effect. This model did not converge, indicating that we were trying to make inferences on too many variables given the data. Because we did not have a-priori hypotheses about recognition rates in relation to specific emotions, we removed Emotion as a factor from the model to reach convergence (we report emotion-level analyses separately in Supplementary Tables 11-14). To assess the significance of fixed effects and their interactions, we then conducted a Type III ANOVA on the fitted model using Wald's chi-square tests.

We found significant main effects of Expression Type ($\chi(1) = 240.74$, $p < .001$), Expressor Culture ($\chi(1) = 79.94$, $p < .001$), and Perceiver Culture ($\chi(1) = 8.21$, $p = .004$), as well as significant interactions between Expression Type and Expressor Culture ($\chi(1) = 9.07$, $p = .003$) and between Expressor Culture and Perceiver Culture ($\chi(1) = 17.20$, $p < .001$). To directly test our hypotheses, we then conducted follow-up analyses to separately test whether there was an ingroup advantage for posed and spontaneous expressions.

Posed Expressions

We performed a mixed logistic regression with trial-level Accuracy for posed expressions as the outcome variable, Expressor Culture and Perceiver Culture as fixed effects, and Perceiver ID as a random effect. Running a Type 3 ANOVA on the logistic model revealed significant main effects of Expressor Culture ($\chi(1) = 80.29$, $p < .001$) and Perceiver Culture ($\chi(1) = 7.66$, $p = .006$), and an interaction between the two ($\chi(1) = 17.27$, $p < .001$). We then ran follow-up logistic regressions testing for an ingroup advantage using the two approaches described above: first holding the culture of the Expressor constant, and then holding Perceiver culture constant.

Holding Expressor Culture Constant. First, we held the culture of the Expressor constant and compared the performance of the Perceiver groups. An ingroup advantage would entail Dutch Perceivers outperforming Chinese Perceivers on Dutch expressions, and Chinese Perceivers outperforming Dutch Perceivers on Chinese expressions. Splitting the data by Expressor Culture, we ran logistic models in which we regressed trial-level

Accuracy on Perceiver Culture, and set Chinese expressions as the intercept. For these models, we report the exponentiated log odds, known as the odds ratio (OR). An odds ratio below 1 indicates a decrease in the odds of accurate recognition, relative to the intercept.

Contrary to our hypothesis, we did not find a mutual ingroup advantage for posed emotional expressions. Instead, we found that Dutch Perceivers outperformed Chinese Perceivers when viewing both Dutch expressions ($OR_{DutchPerceivers}$ = 1.28, 95% CI [1.21, 1.36], p < .001), and Chinese expressions ($OR_{DutchPerceivers}$ = 1.10, 95% CI [1.02, 1.17], p = .008). The odds ratio being larger than 1 indicates that the odds of a correct response is higher for Dutch Perceivers, compared to Chinese Perceivers (set as the intercept). This means that following the first approach (i.e., holding Expressor Culture constant while comparing performance between Dutch and Chinese Perceivers), we found an ingroup advantage with Dutch expressions, but not with Chinese expressions. Per-emotion analyses are reported in Supplementary Table 11.

The finding that Dutch Perceivers outperformed Chinese Perceivers when viewing Chinese expressions contrasts with our hypothesis of an ingroup advantage. We therefore evaluated the relative support in the observed data for the original hypothesis (Chinese Perceivers > Dutch Perceivers) versus the null (Chinese Perceivers <= Dutch Perceivers) using Bayesian inference for informative hypotheses. We used the R package `bain`, version 0.2.11 (Hoijtink et al, 2019), which uses the Bayes factor to evaluate the support for hypotheses in a wide range of models, including mixed effects models. A Bayes factor quantifies this support in terms of the relative likelihood of one hypothesis over the other. For example, $BF_1 = 3$ means the data are 3 times more likely under the alternative hypothesis than the null hypothesis. We found extremely low support for the alternative hypothesis (Chinese Perceivers > Dutch Perceivers) compared to the null (BF_1 = 0.004). The Bayes factor in favour of the null can also be expressed as the reciprocal of BF_1. In this case, we have very strong evidence in favour of the null hypothesis ($BF_0 = 1 / BF_1 = 261.13$) over the ingroup advantage hypothesis.

Holding Perceiver Culture Constant. Next, we tested whether there is an ingroup advantage following the second approach frequently used in the literature. The second approach holds Perceiver Culture constant while comparing the recognition accuracy of expressions from different Expressor groups. In this approach, an ingroup advantage would entail Chinese Perceiveres performing better on Chinese expressions than Dutch expressions, and Dutch Perceivers performing better on Dutch expressions than Chinese expressions.

Again, we did not find a mutual ingroup advantage using this method. Instead, we found that emotions were more accurately recognised from Chinese expressions than from Dutch expressions by both Chinese ($OR_{DutchExpressions}$ = 0.77, 95% CI [0.73, 0.82], p < .001) and Dutch Perceivers ($OR_{DutchExpressions}$ = 0.91, 95% CI [0.86, 0.96], p < .001). The odds ratios being lower than 1 indicate that the odds of a correct response have *decreased* relative to the intercept (Chinese expressions).

This means that the Chinese posed expressions were overall better recognised, regardless of Perceiver Culture. We tested this interpretation using Bayesian inference and found clear support: when holding Perceiver Culture constant, there was strong evidence in favour of an ingroup advantage for Chinese Perceivers (i.e., Chinese expressions > Dutch expressions), BF_1 = 2.29 × 10ˆ13, but strong evidence against an ingroup advantage for Dutch Perceivers (i.e., Dutch expressions > Chinese expressions), BF_1 = 0.0004 or BF_0 = 2525.39. Per-emotion analyses are reported in Supplementary Table 12.

Spontaneous Expressions

To investigate the ingroup advantage in the recognition of spontaneous expressions, we followed the same analytical approach as for posed expressions. We found a significant main effect of Expressor Culture ($\chi(1)$ = 16.80, p < .001), but not Perceiver Culture ($\chi(1)$ = 1.03, p = 0.32), and no interaction between the two ($\chi(1)$ = 3.03, p = .08). We thus did not observe a mutual ingroup advantage for the spontaneous expressions either.

Holding Expressor Culture Constant. Dutch Perceivers outperformed Chinese Perceivers when viewing Dutch stimuli ($OR_{DutchPerceivers}$ = 1.11, 95% CI [1.05, 1.18], p < .001), with the data providing strong evidence in favour of an ingroup advantage for Dutch stimuli (BF_1 = 2679.54). However, when viewing

Chinese stimuli, recognition accuracy of Dutch and Chinese Perceivers did not significantly differ from each other ($OR_{DutchPerceivers} = 1.03$, 95% CI [0.97, 1.09], p = 0.29); the data provided moderate support for the null hypothesis that Chinese Perceivers do not perform better than Dutch Perceivers when viewing Chinese stimuli ($BF_0 = 5.91$). This means that when holding Expressor Culture constant, we found an ingroup advantage for Dutch expressions, but not for Chinese expressions. Per-emotion analyses are reported in Supplementary Table 13 and visualised in Supplementary Figure 2.

Holding Perceiver Culture Constant. We found that Chinese Perceivers were better at recognising emotions from Chinese expressions than from Dutch expressions ($OR_{DutchExpressions} = 0.88$, 95% CI [0.83, 0.94], $p < .001$), whereas the Dutch perceivers did not perform differently with regard to Chinese and Dutch expressions ($OR_{DutchExpressions} = 0.95$, 95% CI [0.89, 1.01], p = 0.08). Bayesian analyses yielded evidence for an ingroup advantage for Chinese Perceivers ($BF_1 = 48027.17$), but evidence against an ingroup advantage for Dutch Perceivers ($BF_0 = 23.08$).

Supplementary Tables

English	*Dutch*	*Chinese*
Anger	Boosheid	气愤
Disgust	Walging	恶心
Fear	Angst	害怕
Sadness	Verdriet	悲伤
Compassion	Medelijden	同情
Love	Liefde	爱
Joy	Plezier	喜悦
Pride	Trots	骄傲

Supplementary Table 1. Emotion labels used in the study, alongside their Dutch and Chinese translations.

	Responses							
Emotions	anger	compassion	disgust	fear	joy	love	pride	sadness
anger	**0.42**	0.09	0.2	0.06	0.04	0.02	0.03	0.12
compassion	0.12	0.17	0.17	0.1	0.09	0.06	0.06	**0.23**
disgust	0.12	0.1	**0.4**	0.09	0.11	0.04	0.05	0.1
fear	0.13	0.12	0.18	**0.25**	0.1	0.04	0.07	0.1
joy	0.02	0.02	0.02	0.02	**0.67**	0.08	0.12	0.03
love	0.03	0.06	0.05	0.04	**0.44**	0.18	0.16	0.04
pride	0.04	0.07	0.05	0.05	**0.47**	0.1	0.19	0.04
sadness	0.11	0.12	0.12	0.11	0.06	0.03	0.04	**0.4**

Supplementary Table 2. Confusion matrix demonstrating the proportion of guesses per emotion when Dutch perceivers judged Dutch posed stimuli. Underlined numbers indicate the correct response for each emotion, bolded numbers indicated the most frequently selected response.

	Responses							
Emotions	anger	compassion	disgust	fear	joy	love	pride	sadness
anger	**0.21**	0.2	0.18	0.1	0.07	0.04	0.1	0.1
compassion	0.1	**0.22**	0.17	0.12	0.12	0.05	0.11	0.12
disgust	0.11	0.13	**0.33**	0.07	0.14	0.06	0.1	0.06
fear	0.16	**0.17**	**0.17**	0.14	0.1	0.05	0.09	0.11
joy	0.03	0.06	0.05	0.05	**0.47**	0.13	0.17	0.03
love	0.04	0.08	0.05	0.05	**0.42**	0.14	0.18	0.04
pride	0.04	0.1	0.08	0.05	**0.38**	0.1	0.21	0.04
sadness	0.13	0.19	0.16	0.14	0.07	0.04	0.07	**0.21**

Supplementary Table 3. Confusion matrix demonstrating the proportion of guesses per emotion when Dutch perceivers judged Dutch spontaneous stimuli. Underlined numbers indicate the correct response for each emotion, bolded numbers indicated the most frequently selected response.

	Responses							
Emotions	anger	compassion	disgust	fear	joy	love	pride	sadness
anger	**0.31**	0.1	0.14	0.16	0.07	0.02	0.06	0.14
compassion	0.12	0.18	0.12	0.19	0.07	0.04	0.06	**0.22**
disgust	0.14	0.08	**0.39**	0.12	0.07	0.03	0.03	0.13
fear	0.08	0.09	0.15	**0.43**	0.08	0.03	0.06	0.08
joy	0.02	0.03	0.03	0.04	**0.6**	0.09	0.16	0.02
love	0.04	0.08	0.04	0.08	**0.35**	0.22	0.15	0.04
pride	0.05	0.08	0.06	0.08	**0.31**	0.06	**0.31**	0.05
sadness	0.12	0.1	0.11	0.16	0.05	0.02	0.04	**0.4**

Supplementary Table 4. Confusion matrix demonstrating the proportion of guesses per emotion when Dutch perceivers judged Chinese posed stimuli. Underlined numbers indicate the correct response for each emotion, bolded numbers indicated the most frequently selected response.

Emotions	Responses							
	anger	compassion	disgust	fear	joy	love	pride	sadness
anger	0.12	0.15	0.11	**0.16**	0.13	0.05	0.15	0.13
compassion	0.1	0.17	0.14	**0.21**	0.1	0.04	0.1	0.14
disgust	0.15	0.1	**0.41**	0.11	0.06	0.02	0.05	0.09
fear	0.08	0.17	0.13	**0.27**	0.1	0.03	0.09	0.12
joy	0.03	0.05	0.04	0.06	**0.54**	0.06	0.18	0.04
love	0.05	0.13	0.09	0.11	**0.32**	0.06	0.17	0.07
pride	0.04	0.09	0.06	0.08	**0.41**	0.05	0.22	0.04
sadness	0.11	0.14	0.13	0.2	0.08	0.03	0.07	**0.22**

Supplementary Table 5. Confusion matrix demonstrating the proportion of guesses per emotion when Dutch perceivers judged Chinese spontaneous stimuli. Underlined numbers indicate the correct response for each emotion, bolded numbers indicated the most frequently selected response.

Emotions	Responses							
	anger	compassion	disgust	fear	joy	love	pride	sadness
anger	**0.28**	0.11	0.09	0.14	0.14	0.03	0.05	0.17
compassion	0.12	0.22	0.05	0.15	0.12	0.05	0.05	**0.24**
disgust	0.12	0.1	**0.32**	0.11	0.14	0.03	0.04	0.14
fear	0.09	0.13	0.09	**0.34**	0.15	0.04	0.07	0.09
joy	0.03	0.04	0.02	0.03	**0.74**	0.05	0.07	0.03
love	0.02	0.08	0.02	0.08	**0.51**	0.17	0.07	0.04
pride	0.05	0.09	0.03	0.06	**0.47**	0.05	0.21	0.04
sadness	0.1	0.13	0.06	0.16	0.08	0.03	0.03	**0.41**

Supplementary Table 6. Confusion matrix demonstrating the proportion of guesses per emotion when Chinese perceivers judged Chinese posed stimuli. Underlined numbers indicate the correct response for each emotion, bolded numbers indicated the most frequently selected response.

Emotions	Responses							
	anger	compassion	disgust	fear	joy	love	pride	sadness
anger	0.14	**0.2**	0.05	0.11	**0.2**	0.06	0.11	0.13
compassion	0.11	**0.23**	0.05	0.17	0.17	0.06	0.09	0.13
disgust	0.16	0.16	**0.26**	0.12	0.1	0.02	0.05	0.13
fear	0.08	**0.23**	0.04	0.2	0.19	0.06	0.08	0.13
joy	0.03	0.05	0.02	0.04	**0.7**	0.05	0.07	0.03
love	0.05	0.16	0.03	0.08	**0.44**	0.07	0.11	0.05
pride	0.04	0.11	0.02	0.06	**0.54**	0.06	0.12	0.06
sadness	0.09	0.22	0.05	0.16	0.14	0.05	0.06	**0.23**

Supplementary Table 7. Confusion matrix demonstrating the proportion of guesses per emotion when Chinese perceivers judged Chinese spontaneous stimuli. Underlined numbers indicate the correct response for each emotion, bolded numbers indicated the most frequently selected response.

Emotions	Responses							
	anger	compassion	disgust	fear	joy	love	pride	sadness
anger	**0.28**	0.11	0.16	0.08	0.08	0.03	0.05	0.21
compassion	0.11	0.19	0.07	0.09	0.15	0.04	0.06	**0.29**
disgust	0.14	0.11	**0.23**	0.11	0.17	0.03	0.04	0.18
fear	0.14	0.16	0.07	0.16	**0.17**	0.05	0.07	**0.17**
joy	0.02	0.03	0.02	0.02	**0.77**	0.05	0.07	0.03
love	0.03	0.08	0.03	0.04	**0.58**	0.1	0.09	0.05
pride	0.04	0.09	0.03	0.03	**0.55**	0.06	0.14	0.05
sadness	0.11	0.16	0.07	0.07	0.1	0.04	0.05	**0.39**

Supplementary Table 8. Confusion matrix demonstrating the proportion of guesses per emotion when Chinese perceivers judged Dutch posed stimuli. Underlined numbers indicate the correct response for each emotion, bolded numbers indicated the most frequently selected response.

Emotions	Responses							
	anger	compassion	disgust	fear	joy	love	pride	sadness
anger	**0.24**	0.21	0.07	0.08	0.12	0.05	0.07	0.16
compassion	0.11	**0.26**	0.05	0.09	0.15	0.08	0.1	0.17
disgust	0.13	0.18	0.14	0.07	**0.25**	0.05	0.07	0.1
fear	0.15	**0.23**	0.06	0.09	0.15	0.06	0.1	0.17
joy	0.04	0.1	0.03	0.03	**0.57**	0.06	0.13	0.05
love	0.03	0.1	0.03	0.03	**0.55**	0.09	0.11	0.05
pride	0.05	0.13	0.03	0.04	**0.48**	0.09	0.13	0.05
sadness	0.12	0.22	0.05	0.1	0.12	0.06	0.07	**0.26**

Supplementary Table 9. Confusion matrix demonstrating the proportion of guesses per emotion when Chinese perceivers judged Dutch spontaneous stimuli. Underlined numbers indicate the correct response for each emotion, bolded numbers indicated the most frequently selected response.

Type	Perceiver	Expressor	Prop. Correct	SD
posed	CN	CN	0.34	0.15
		NL	0.28	0.12
	NL	CN	0.36	0.14
		NL	0.34	0.13
spontaneous	CN	CN	0.25	0.11
		NL	0.22	0.10
	NL	CN	0.25	0.10
		NL	0.24	0.11

Supplementary Table 10. Recognition accuracy across factors. Recognition accuracy was calculated as the proportion of correct responses for each participant, averaged.

Expressor	Emotion	Perceiver	Estimate	OR	Std. Error	Statistic	p	Adj. p	
CN	anger	Chinese (Intercept)	-1.01	0.36	0.07	-14.16	0.00	0.00	***
		Dutch	0.13	1.14	0.09	1.45	0.15	1.00	
	compassion	Chinese (Intercept)	-1.47	0.23	0.09	-16.06	0.00	0.00	***
		Dutch	-0.30	0.74	0.11	-2.72	0.01	0.41	
	disgust	Chinese (Intercept)	-0.90	0.41	0.07	-12.19	0.00	0.00	***
		Dutch	0.37	1.45	0.10	3.93	0.00	0.01	*
	fear	Chinese (Intercept)	-0.76	0.47	0.07	-10.52	0.00	0.00	***
		Dutch	0.43	1.54	0.10	4.49	0.00	0.00	***
	joy	Chinese (Intercept)	1.33	3.78	0.09	14.21	0.00	0.00	***
		Dutch	-0.79	0.45	0.12	-6.84	0.00	0.00	***
	love	Chinese (Intercept)	-1.95	0.14	0.11	-17.10	0.00	0.00	***
		Dutch	0.40	1.49	0.12	3.33	0.00	0.06	
	pride	Chinese (Intercept)	-1.40	0.25	0.08	-17.32	0.00	0.00	***
		Dutch	0.57	1.76	0.09	6.09	0.00	0.00	***
	sadness	Chinese (Intercept)	-0.42	0.66	0.06	-6.57	0.00	0.00	***
		Dutch	-0.03	0.97	0.09	-0.39	0.70	1.00	
NL	***anger***	Chinese (Intercept)	-1.04	0.35	0.07	-14.27	0.00	0.00	***
		Dutch	0.69	1.99	0.09	7.46	0.00	0.00	***
	compassion	Chinese (Intercept)	-1.64	0.19	0.10	-16.83	0.00	0.00	***
		Dutch	-0.13	0.87	0.11	-1.22	0.22	1.00	
	disgust	Chinese (Intercept)	-1.37	0.25	0.08	-16.50	0.00	0.00	***
		Dutch	0.91	2.48	0.10	9.17	0.00	0.00	***
	fear	Chinese (Intercept)	-1.90	0.15	0.11	-18.02	0.00	0.00	***
		Dutch	0.62	1.86	0.11	5.62	0.00	0.00	***
	joy	Chinese (Intercept)	1.55	4.71	0.10	15.54	0.00	0.00	***
		Dutch	-0.62	0.54	0.12	-5.34	0.00	0.00	***
	love	Chinese (Intercept)	-2.75	0.06	0.16	-17.51	0.00	0.00	***
		Dutch	0.82	2.27	0.14	5.80	0.00	0.00	***
	pride	Chinese (Intercept)	-1.99	0.14	0.11	-18.05	0.00	0.00	***
		Dutch	0.37	1.45	0.11	3.34	0.00	0.05	
	sadness	Chinese (Intercept)	-0.49	0.61	0.06	-7.74	0.00	0.00	***
		Dutch	0.04	1.04	0.09	0.51	0.61	1.00	

Supplementary Table 11. In-group Advantage for Posed Stimuli with Expressor Culture Held Constant This table shows the results of mixed-effects logistic regressions, regressing binary Recognition Accuracy on Perceiver Culture, with Participant ID treated as a random effect. Chinese Perceivers were set as the intercept, while the estimate associated with Dutch Perceivers indicates the difference between the two Perceiver groups. We applied a Bonferroni correction to account for multiple comparisons (Adj. p). Significance stars refer to these corrected values. We have italicised and bolded the emotions where we observe an effect in the direction predicted by the in-group advantage account. (CN = Chinese, NL = Dutch)

Perceiver	Emotion	Expressor	Estimate	OR	Std. Error	Statistic	p	Adj. p	
CN	anger	Chinese (Intercept)	-1.02	0.36	0.07	-14.90	0.00	0.00	***
		Dutch	-0.01	0.99	0.09	-0.16	0.87	1.00	
	compassion	Chinese (Intercept)	-1.38	0.25	0.07	-18.38	0.00	0.00	***
		Dutch	-0.17	0.85	0.09	-1.77	0.08	1.00	
	disgust	Chinese (Intercept)	-0.94	0.39	0.08	-12.39	0.00	0.00	***
		Dutch	-0.52	0.59	0.09	-5.68	0.00	0.00	***
	fear	Chinese (Intercept)	-0.74	0.48	0.07	-10.81	0.00	0.00	***
		Dutch	-1.12	0.32	0.10	-11.56	0.00	0.00	***
	joy	Chinese (Intercept)	1.39	4.02	0.09	14.65	0.00	0.00	***
		Dutch	0.24	1.27	0.10	2.43	0.02	0.96	
	love	Chinese (Intercept)	-1.91	0.15	0.10	-18.25	0.00	0.00	***
		Dutch	-0.78	0.46	0.12	-6.41	0.00	0.00	***
	pride	Chinese (Intercept)	-1.43	0.24	0.08	-18.22	0.00	0.00	***
		Dutch	-0.51	0.60	0.10	-5.08	0.00	0.00	***
	sadness	Chinese (Intercept)	-0.42	0.66	0.06	-6.61	0.00	0.00	***
		Dutch	-0.08	0.92	0.08	-0.98	0.33	1.00	
NL	***anger***	Chinese (Intercept)	-0.86	0.42	0.06	-13.74	0.00	0.00	***
		Dutch	0.52	1.68	0.08	6.58	0.00	0.00	***
	compassion	Chinese (Intercept)	-1.68	0.19	0.08	-20.08	0.00	0.00	***
		Dutch	-0.01	0.99	0.10	-0.13	0.89	1.00	
	disgust	Chinese (Intercept)	-0.50	0.61	0.06	-8.14	0.00	0.00	***
		Dutch	0.05	1.05	0.08	0.69	0.49	1.00	
	fear	Chinese (Intercept)	-0.31	0.73	0.06	-5.18	0.00	0.00	***
		Dutch	-0.89	0.41	0.08	-10.64	0.00	0.00	***
	joy	Chinese (Intercept)	0.52	1.69	0.07	7.08	0.00	0.00	***
		Dutch	0.39	1.47	0.09	4.53	0.00	0.00	***
	love	Chinese (Intercept)	-1.41	0.24	0.08	-17.75	0.00	0.00	***
		Dutch	-0.34	0.71	0.10	-3.56	0.00	0.02	*
	pride	Chinese (Intercept)	-0.82	0.44	0.06	-13.32	0.00	0.00	***
		Dutch	-0.70	0.50	0.09	-7.96	0.00	0.00	***
	sadness	Chinese (Intercept)	-0.43	0.65	0.06	-7.44	0.00	0.00	***
		Dutch	0.00	1.00	0.08	0.02	0.98	1.00	

Supplementary Table 12. In-group Advantage for Posed Stimuli with Perceiver Culture Held Constant This table shows the results of mixed-effects logistic regressions, regressing binary Recognition Accuracy on Expressor Culture, with Participant ID treated as a random effect. Chinese stimuli were set as the intercept, while the estimate associated with Dutch stimuli indicates the difference between the two stimuli cultures. We applied a Bonferroni correction to account for multiple comparisons (Adj. p). Significance stars refer to these corrected values. We have italicised and bolded the emotions where we observe an effect in the direction predicted by the in-group advantage account. (CN = Chinese, NL = Dutch)

Expressor	Emotion	Perceiver	Estimate	OR	Std. Error	Statistic	p	Adj. p	
CN	anger	Chinese (Intercept)	-1.92	0.15	0.11	-16.79	0.00	0.00	***
		Dutch	-0.26	0.77	0.12	-2.20	0.03	1.00	
	compassion	Chinese (Intercept)	-1.31	0.27	0.08	-15.77	0.00	0.00	***
		Dutch	-0.43	0.65	0.10	-4.25	0.00	0.00	***
	disgust	Chinese (Intercept)	-1.03	0.36	0.06	-17.52	0.00	0.00	***
		Dutch	0.66	1.94	0.08	8.39	0.00	0.00	***
	fear	Chinese (Intercept)	-1.52	0.22	0.09	-17.33	0.00	0.00	***
		Dutch	0.45	1.56	0.10	4.52	0.00	0.00	***
	joy	Chinese (Intercept)	1.03	2.80	0.08	12.98	0.00	0.00	***
		Dutch	-0.84	0.43	0.10	-8.16	0.00	0.00	***
	love	Chinese (Intercept)	-3.08	0.05	0.21	-14.38	0.00	0.00	***
		Dutch	-0.11	0.90	0.17	-0.65	0.51	1.00	
	pride	Chinese (Intercept)	-2.15	0.12	0.12	-18.48	0.00	0.00	***
		Dutch	0.75	2.11	0.11	6.60	0.00	0.00	***
	sadness	Chinese (Intercept)	-1.31	0.27	0.08	-16.10	0.00	0.00	***
		Dutch	-0.04	0.96	0.10	-0.45	0.66	1.00	
NL	anger	Chinese (Intercept)	-1.27	0.28	0.08	-16.02	0.00	0.00	***
		Dutch	-0.13	0.88	0.10	-1.31	0.19	1.00	
	compassion	Chinese (Intercept)	-1.17	0.31	0.08	-14.73	0.00	0.00	***
		Dutch	-0.26	0.77	0.10	-2.64	0.01	0.53	
	disgust	Chinese (Intercept)	-1.98	0.14	0.10	-19.31	0.00	0.00	***
		Dutch	1.18	3.25	0.11	10.86	0.00	0.00	***
	fear	Chinese (Intercept)	-2.36	0.09	0.14	-17.19	0.00	0.00	***
		Dutch	0.48	1.61	0.12	3.96	0.00	0.00	***
	joy	Chinese (Intercept)	0.31	1.36	0.06	4.95	0.00	0.00	***
		Dutch	-0.43	0.65	0.09	-4.96	0.00	0.00	***
	love	Chinese (Intercept)	-2.46	0.09	0.14	-17.38	0.00	0.00	***
		Dutch	0.49	1.63	0.13	3.87	0.00	0.01	*
	pride	Chinese (Intercept)	-2.27	0.10	0.13	-17.97	0.00	0.00	***
		Dutch	0.67	1.96	0.12	5.39	0.00	0.00	***
	sadness	Chinese (Intercept)	-1.20	0.30	0.08	-14.68	0.00	0.00	***
		Dutch	-0.32	0.72	0.10	-3.17	0.00	0.10	

Supplementary Table 13. In-group Advantage for Spontaneous Stimuli with Expressor Culture Held Constant This table shows the results of mixed-effects logistic regressions, regressing binary Recognition Accuracy on Perceiver Culture, with Participant ID treated as a random effect. Chinese Perceivers were set as the intercept, while the estimate associated with Dutch Perceivers indicates the difference between the two Perceiver groups. We applied a Bonferroni correction to account for multiple comparisons (Adj. p). Significance stars refer to these corrected values. We have italicised and bolded the emotions where we observe an effect in the direction predicted by the in-group advantage account. (CN = Chinese, NL = Dutch)

Perceiver	Emotion	Expressor	Estimate	OR	Std. Error	Statistic	p	Adj. p	
CN	anger	Chinese (Intercept)	-1.95	0.14	0.09	-21.17	0.00	0.00	***
		Dutch	0.65	1.91	0.10	6.43	0.00	0.00	***
	compassion	Chinese (Intercept)	-1.35	0.26	0.08	-17.49	0.00	0.00	***
		Dutch	0.18	1.20	0.09	2.01	0.04	1.00	
	disgust	Chinese (Intercept)	-1.13	0.32	0.07	-15.86	0.00	0.00	***
		Dutch	-0.83	0.44	0.10	-8.34	0.00	0.00	***
	fear	Chinese (Intercept)	-1.46	0.23	0.08	-18.27	0.00	0.00	***
		Dutch	-0.93	0.39	0.11	-8.19	0.00	0.00	***
	joy	Chinese (Intercept)	1.00	2.71	0.07	13.74	0.00	0.00	***
		Dutch	-0.67	0.51	0.08	-7.97	0.00	0.00	***
	love	Chinese (Intercept)	-2.90	0.05	0.15	-19.65	0.00	0.00	***
		Dutch	0.37	1.45	0.14	2.63	0.01	0.54	
	pride	Chinese (Intercept)	-2.31	0.10	0.11	-20.28	0.00	0.00	***
		Dutch	0.09	1.09	0.12	0.75	0.45	1.00	
	sadness	Chinese (Intercept)	-1.34	0.26	0.08	-17.55	0.00	0.00	***
		Dutch	0.17	1.19	0.09	1.92	0.06	1.00	
NL	***anger***	Chinese (Intercept)	-2.12	0.12	0.10	-22.23	0.00	0.00	***
		Dutch	0.75	2.12	0.10	7.24	0.00	0.00	***
	compassion	Chinese (Intercept)	-1.66	0.19	0.08	-21.01	0.00	0.00	***
		Dutch	0.34	1.40	0.09	3.62	0.00	0.02	*
	disgust	Chinese (Intercept)	-0.39	0.67	0.06	-6.96	0.00	0.00	***
		Dutch	-0.37	0.69	0.08	-4.73	0.00	0.00	***
	fear	Chinese (Intercept)	-1.02	0.36	0.07	-15.67	0.00	0.00	***
		Dutch	-0.87	0.42	0.10	-9.05	0.00	0.00	***
	joy	Chinese (Intercept)	0.18	1.20	0.06	2.90	0.00	0.24	
		Dutch	-0.31	0.73	0.08	-3.98	0.00	0.00	***
	love	Chinese (Intercept)	-2.88	0.06	0.14	-21.04	0.00	0.00	***
		Dutch	0.94	2.56	0.13	7.08	0.00	0.00	***
	pride	Chinese (Intercept)	-1.40	0.25	0.08	-18.47	0.00	0.00	***
		Dutch	-0.04	0.96	0.09	-0.48	0.63	1.00	
	sadness	Chinese (Intercept)	-1.32	0.27	0.07	-18.40	0.00	0.00	***
		Dutch	-0.10	0.91	0.09	-1.10	0.27	1.00	

Supplementary Table 14. In-group Advantage for Spontaneous Stimuli with Perceiver Culture Held Constant This table shows the results of mixed-effects logistic regressions, regressing binary Recognition Accuracy on Expressor Culture, with Participant ID treated as a random effect. Chinese stimuli were set as the intercept, while the estimate associated with Dutch stimuli indicates the difference between the two stimuli cultures. We applied a Bonferroni correction to account for multiple comparisons (Adj. p). Significance stars refer to these corrected values. We have italicised and bolded the emotions where we observe an effect in the direction predicted by the in-group advantage account. (CN = Chinese, NL = Dutch)

Supplementary Figures

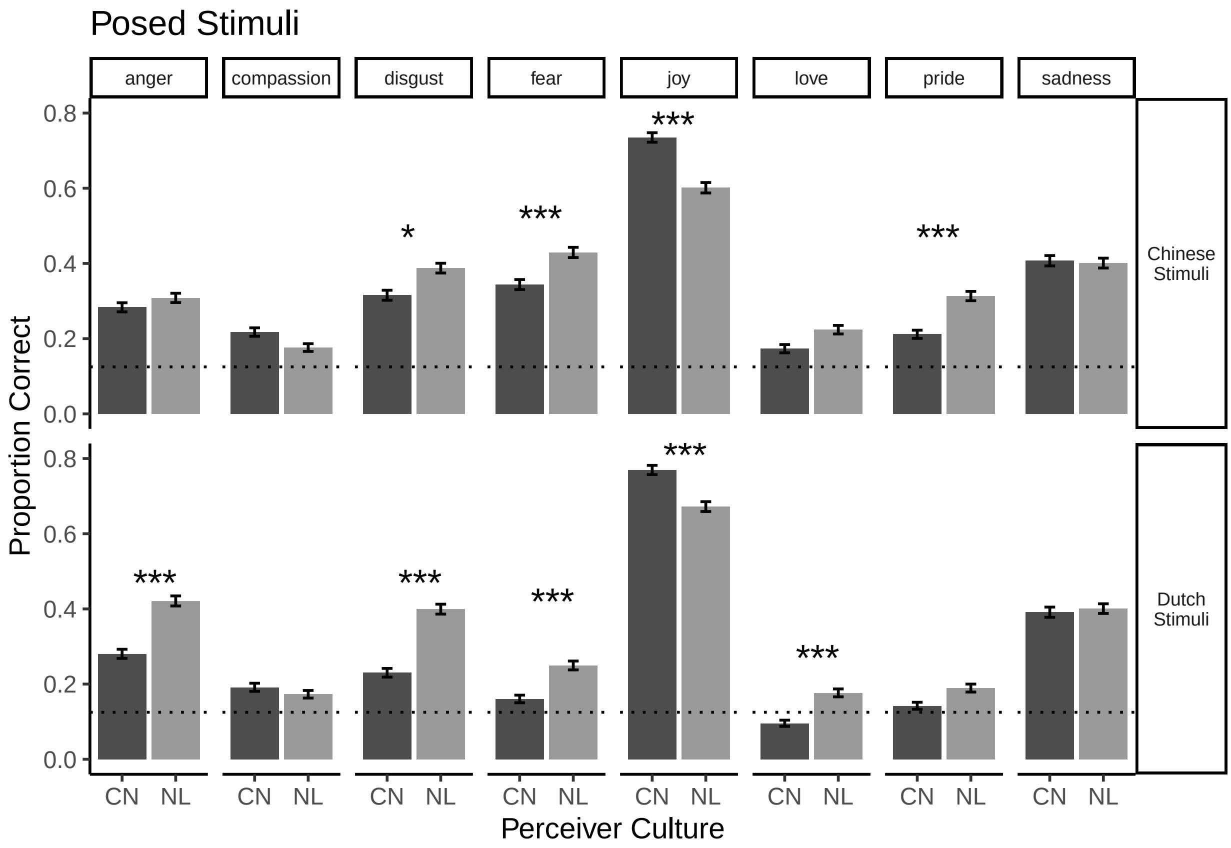

Supplementary Figure 1. In-group advantage across emotions for posed stimuli, holding Expressor Culture constant and comparing the performance of Dutch (NL) and Chinese (CN) Perceivers. The bars indicate recognition accuracy as a proportion of correct responses across all trials, averaged across participants. Error bars reflect standard errors. The dotted line denotes chance-level recognition (1/8 = .125). Significance stars refer to the mixed logistic regressions reported in Supplementary Table 1. *** $p < .001$, ** $p < .01$, * $p < .05$.

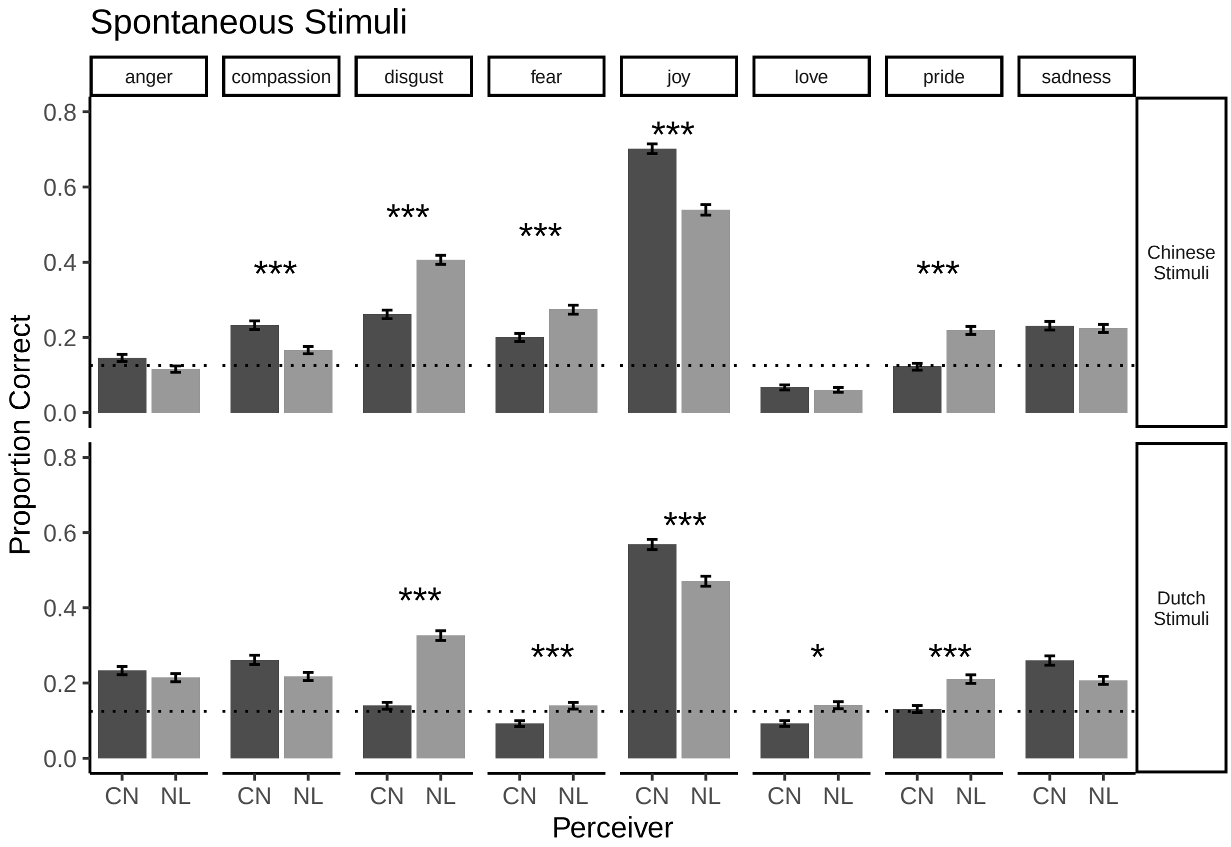

Supplementary Figure 2. In-group advantage across emotions for spontaneous stimuli, holding Expressor Culture constant and comparing the performance of Dutch (NL) and Chinese (CN) Perceivers. The bars indicate recognition accuracy as a proportion of correct responses across all trials, averaged across participants. Error bars reflect standard errors. The dotted line denotes chance-level recognition (1/8 = .125). Significance stars refer to the mixed logistic regressions reported in Supplementary Table 3. *** p < .001, ** p < .01, * p < .05.
